# Urgent hyperbaric oxygen therapy for suicidal carbon monoxide poisoning: from a preliminary survey to a proposal for an integrated somatic-psychiatric protocol

**DOI:** 10.1186/s12245-020-00321-w

**Published:** 2020-12-02

**Authors:** Alessandra Costanza, Julia Ambrosetti, Philippe Spagnoli, Andrea Amerio, Andrea Aguglia, Gianluca Serafini, Mario Amore, Guido Bondolfi, François Sarasin, Rodrigue Pignel

**Affiliations:** 1 Department of Psychiatry, Faculty of Medicine, University of Geneva (UNIGE), Geneva, Switzerland; 2 Department of Psychiatry, ASO Santi Antonio e Biagio e Cesare Arrigo Hospital, Alessandria, Italy; 3 Emergency Department and Psychiatry Department, Emergency Psychiatric Unit (UAUP), Geneva University Hospitals (HUG), Geneva, Switzerland; 4 Emergency Department, Hyperbaric Medicine Unit, Geneva University Hospitals (HUG), Geneva, Switzerland; 5 Department of Neuroscience, Rehabilitation, Ophthalmology, Genetics, Maternal and Child Health (DINOGMI), Section of Psychiatry, University of Genoa, Genoa, Italy; 6 IRCCS Ospedale Policlinico San Martino, Genoa, Italy; 7 Department of Psychiatry, Tufts University, Boston, USA; 8 Department of Psychiatry, Service of Liaison Psychiatry and Crisis Intervention (SPLIC), Geneva University Hospitals (HUG), Geneva, Switzerland; 9 Emergency Department, Emergency Medicine Unit, Geneva University Hospitals (HUG), Geneva, Switzerland

**Keywords:** Hyperbaric medicine, Emergency medicine, Emergency psychiatry, Risk management, Suicide, Suicidal behavior, Suicide attempt, Intentional carbon monoxide intoxication

## Abstract

A considerable number of patients who made a carbon monoxide (CO) suicidal attempt are treated with urgent hyperbaric oxygen therapy (HBOT). For these patients at potential persistent risk of suicide, the hyperbaric chamber is a dangerous environment and their management a complex challenge for the Emergency Department (ED) and Hyperbaric Medicine Unit (UMH) teams. We aimed to (1) identify cases of intentional CO poisoning treated with urgent HBOT in the UMH of the University Hospitals of Geneva (HUG) during 2011–2018 and (2) test a proposed operational and integrated somatic-psychiatric protocol based on acquired experience. A total of 311 patients with CO poisoning were treated using urgent HBOT, for which poisoning was assumed suicidal in 40 patients (12.9%). This percentage appears greater than in other European countries. Both the excess of cases of intentional CO poisonings and difficulties encountered in their management resulted in the implementation of an operational and integrated somatic-psychiatric protocol addressing the entire patient’s clinical trajectory, from the admission at ED-HUG to the treatment at the UMH-HUG. The established institutional protocol includes (1) clinical evaluation, (2) suicide risk assessment, and (3) safety measures. This is the first report—at our best knowledge—of a protocol detailing a practical procedure algorithm and focusing on multidisciplinary and mutual collaboration between the medical-nursing teams at the ED, psychiatric ED, and UMH. Improvements in patient’s safety and care team’s sense of security were observed. In conclusion, the opportunity to refer to a standardized protocol was beneficial in that it offers both reduced risks for suicidal patients and reduced stress for care teams operating in very acute and complex situations. Further studies are needed.

## Background

Carbon monoxide (CO) is one of the leading causes of morbidity and mortality of toxic origin [1]. About 40–70% of American CO poisonings are intentional, whereas the majority of European cases are accidental [2]. Although suicide is the fourth leading cause of early death in Switzerland, CO-specific epidemiologic data is sparse [3, 4, 5]. 

Severe CO poisoning can result in delayed encephalopathy, causing long-term neuropsychiatric symptoms, such as cognitive decline, personality changes, and affective disorders [6]. Although urgent hyperbaric oxygen therapy (HBOT) is not generally considered superior to normobaric oxygen therapy to prevent these sequelae [7], a number of CO poisonings, including intentional poisonings, are treated with HBOT.

**Fig. 1:**
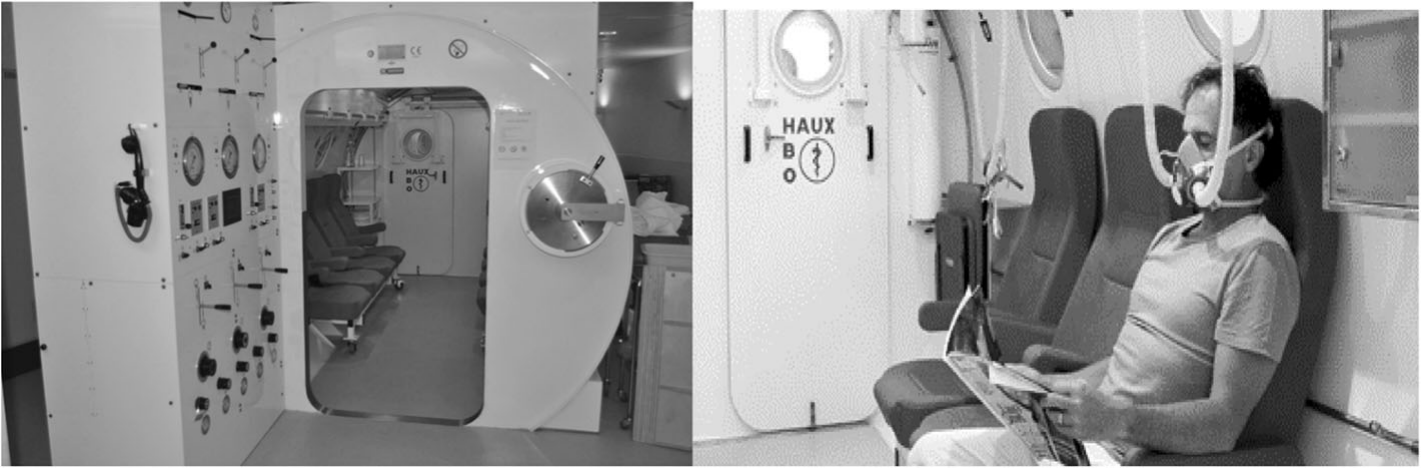
The interior of a multiplace hyperbaric chamber

CO poisoning represents a challenge for the staff of the Department of Emergency (ED). Among suicidal patients, this diagnosis is complicated by frequent simultaneous consumption of alcohol and drugs. Although the risk of repeating a suicide attempted in a hyperbaric chamber is low (especially for monoplace chambers versus multiplace chambers), the hyperbaric chamber is a hazardous environment for these patients, potentially still presenting an acute suicide risk, because of the presence of cables, devices, and other medical equipment (Fig. 1). Finally, in contrast to the standard decompression protocol (requiring at least 15 min), the necessity of applying a faster decompression procedure (requiring 3 min) in cases of acute psychomotor agitation—not rare in these patients—increases the risk of decompression sickness [2, 7, 8].

The aims of this text were to (1) determine if there are sufficient numbers of patients to support adoption of an operational and integrated somatic-psychiatric protocol for patients who made a suicide attempt (SA) by CO poisoning, based on acquired experience, and (2) outline details of this proposed protocol and begin to test its efficacy by implementation in the clinic.

## Methods

### Case identification

Annual numbers of patients with CO poisoning admitted at the ED-HUG and requiring urgent HBOT in the UMH-HUG from January 2011 to December 2018 were retrospectively collected. The percentage of intentional poisoning was calculated to determine whether there were a sufficient number of patients with adverse events to justify adoption of a protocol. Confidential/sensitive personal health information was not collected. As attested in the statement of 17 July 2020, this project did not need to be reviewed by the Geneva Cantonal of the Research Ethics Commission (CCER), because its aims are outside of the scope of the law. This Act applies to research concerning human diseases and concerning the structure and the function of the human body as defined in the Art. 2 of the Human Research Act (HRA) [9].

### Proposed protocol

**Fig. 2:**
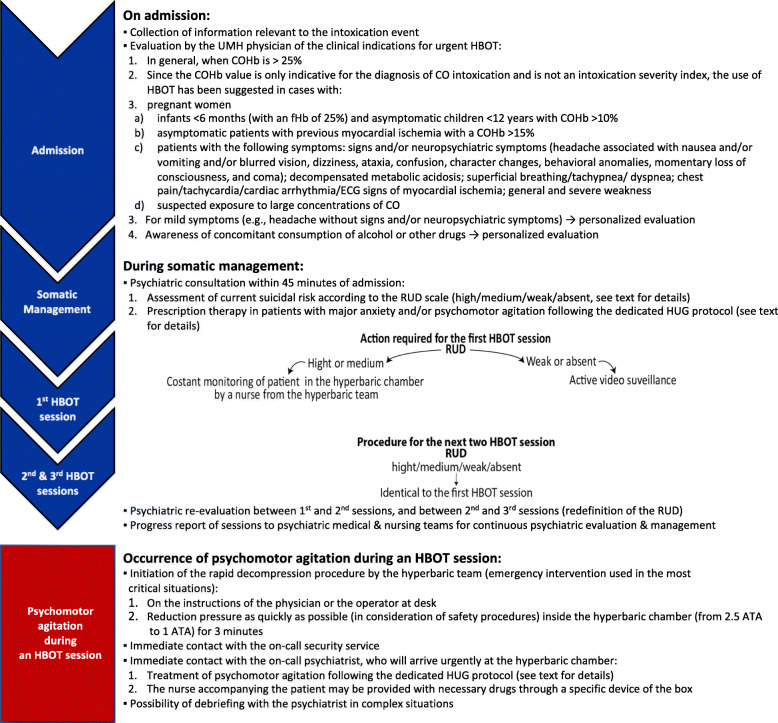
The operational integrated somatic-psychiatric protocol for the management of suicidal patients requiring urgent hyperbaric oxygen treatment. ATA, atmosphere absolute; CO, carbon monoxide; COHb, carboxyhemoglobin; ECG, electrocardiogram; fHb, free hemoglobin; HBOT, hyperbaric oxygen therapy; HUG, University Hospitals of Geneva; RUD, Risk, Urgency, and Dangerousness scale; UMH, Hyperbaric Medicine Unit

Both the excess of cases of intentional CO poisonings and difficulties encountered in their management resulted in the implementation of an operational and integrated somatic-psychiatric protocol addressing the entire patient’s clinical trajectory, from the admission at ED to the treatment at the UMH. This project was facilitated by the geographical proximity in the HUG of the ED, the psychiatric ED, and the UMH. The established protocol includes (1) clinical evaluation, (2) suicide risk assessment, and (3) safety measures (Fig. 2).

Suicide risk was assessed by the Risk, Urgency, and Dangerousness (RUD) scale [10]. Evaluation of these parameters results in four RUD profiles for suicidal behavior (high, medium, weak, absent) with consequential clinical attitudes.

Psychopharmacological indications to be adopted in cases of major anxiety or psychomotor agitation (Fig. 2) follow the dedicated HUG protocol [11]. The latter is based on the consensus statement of the American Association for Emergency Psychiatry [12]. First-line therapy is orally administered 2 mg lorazepam combined with 5–10 mg sublingual olanzapine (2–5 mg haloperidol if olanzapine is unavailable). As a last resort, intramuscular 2 mg lorazepam and 5 mg haloperidol are used (2.5 mg midazolam if lorazepam is unavailable). These regimens can be repeated following a 20–30-min interval in patients with persistent agitation and only following somatic reassessment. Regular supervision is required every 30 min after sedation including monitoring of vital signs (Glasgow Coma Scale, arterial pressure, heart rate, oxygen saturation, blood glucose). Lack of a response should lead to consideration of sedation under airway protection by an anesthetist. These indications should be adapted to the patients’ clinical characteristics, including reducing treatment dosage in the elderly, and the sole or priority use of benzodiazepines in case of documented/suspected pregnancy, alcohol and substance abuse, alcohol and substance withdrawal, concomitant methadone therapy, and prolonged QTc history.

## Results and discussion

A total of 311 patients were treated for CO poisoning over the period 2011–2018. They received hyperbaric oxygen alternating with a 5-min break (to reduce risk of oxygen neurotoxicity) at 2.5 ATA over the course of 3 sessions (2.5, 1.5, and 1.5 h) within the first 24 h. Intentional poisoning was observed in 12.9% (*n* = 40) of cases, with most occurrences in 2012 (23.3%, *n* = 7) and the least in 2017 (7.4%, *n* = 4) (Table 1). The percentage of intentional CO poisoning treated using urgent HBOT appears greater in our sample compared to other European countries. For example, intentional CO poisonings in the UMH of Marseille were estimated at 1% of all treated CO poisonings (Dr. M. Coulange, Centre Hyperbare St. Marguerite, unpublished data). Comparative studies between the UMH various sites inside and outside of Switzerland are needed.

**Table 1:** Accidental and intentional carbon monoxide (CO) poisonings treated using hyperbaric oxygen therapy (HBOT) at the Hyperbaric Medicine Unit of the University Hospitals of Geneva (HUG)

Year	Total CO poisonings	Intentional CO poisonings	
	Patients (*n* = 311)	Patients (*n* = 40)	Percentage (12.9%)
2011	*n* = 22	*n* = 5	22.7%
2012	*n* = 30	*n* = 7	23.3%
2013	*n* = 16	*n* = 2	12.5%
2014	*n* = 48	*n* = 5	10.5%
2015	*n* = 40	*n* = 4	10.0%
2016	*n* = 44	*n* = 4	9.1%
2017	*n* = 54	*n* = 4	7.4%
2018	*n* = 57	*n* = 9	15.8%

Before the implementation of this protocol (years 2011–2013), 14 patients were treated for intentional CO intoxication, which included 4 cases of severe psychomotor agitation requiring a psychiatrist’s intervention. Subsequently, after the implementation of the protocol (years 2014–2018), 26 patients were treated for intentional CO intoxication, which included 2 cases of mild-moderate psychomotor agitation requiring a psychiatrist’s intervention. The risk of committing suicide in a hyperbaric chamber is very low, and this is especially true for monoplace chambers. Nonetheless, we documented 3 cases of a renewed suicide attempt within the multiplace hyperbaric chamber between 2011 and 2013, and no cases afterwards. Although these numbers are not high enough for a rigorous statistical analysis, the trends do suggest that patients may be benefiting from this protocol in terms of reduced severity of psychomotor agitation and lower number of renewed SA.

Improvements in patient’s safety and the UMH team’s sense of security were observed through semi-structured interviews with staff. Before the protocol’s introduction, four cases of severe psychomotor agitation were treated with the intervention of a psychiatrist and there were three cases of a renewed suicide attempt within the hyperbaric chamber. However, at this time, the psychiatrist was not made aware of the risks associated with the hyperbaric chamber nor HBOT procedures. We believe that a more rapid intervention and implementation of the protocol, including administering psychopharmacologic drugs into the chamber, could have negated worsening of the psychomotor agitation. No further incidents occurred after the protocol’s implementation. Concerning subjective impressions of the UMH team, globally, the opportunity to refer to a clear multidisciplinary standardized protocol during acute and complex situations was reported. Some examples of responses of operators during semi-structured interviews were as follows: they could dispose of clear indication during emergencies; when they called for the psychiatrist, the latter was aware of this protocol and he could immediately operate; they prepared with the aid of the psychiatrist a stock of potential necessary psychopharmacological drugs, so the latter were immediately ready when necessary; even if an accident occurred, they know that they follow institutional procedures; finally, they know that they can benefit from debriefing seances with the psychiatrist for complex situations.

These observations suggest that an institutional somatic-psychiatric protocol can be beneficial as it offers both a decreased risk for these suicidal patients and reduced stress for the care teams. Practice recommendations in the diagnosis, management, and prevention of CO poisoning are available [7, 13], but—at our best knowledge—this is the first report of a multidisciplinary protocol detailing clinical procedures, suicide risk assessment, and safety measures focusing on mutual collaboration between the medical-nursing teams at the ED, psychiatric ED, and UMH.

## Limitations

Our work has several limitations. First, the lack of socio-demographic and diagnostic information in this preliminary survey did not allow for identification of confounding factors. Second, formal comparisons between the UMH various sites inside and outside of Switzerland were not made. Third, the only empirical observations on the outcomes of the proposed protocol in the absence of statistical data support did not permit objective measures of improvements in utility and safeness [14].

## Conclusions

In conclusion, we have formulated a multidisciplinary coordinated approach to address SA made by CO poisoning. The descriptive data collected thus far suggests that a somatic-psychiatric protocol can be helpful as it offers both a decreased risk for the suicidal patient and reduced stress for the care teams. It will serve as a framework for future quantitative studies.

## Data Availability

The datasets generated for this study are available on request to the corresponding author.

## Abbreviations

CO: Carbon monoxide
ED: Emergency Department
HBOT: Hyperbaric oxygen therapy
HUG: University Hospitals of Geneva
QTc: Corrected QT interval
RUD: Risk, Urgency, and Dangerousness
UMH: Hyperbaric Medicine Unit
